# Robot-Assisted Ultra-Low Anterior Resection for Rectal Neuroendocrine Tumors after Severe Perineal Tears: A Case Report

**DOI:** 10.70352/scrj.cr.24-0012

**Published:** 2025-02-01

**Authors:** Kenji Baba, Masumi Wada, Naoki Kuroshima, Yuto Hozaka, Daisaku Kamiimabeppu, Masataka Shimonosono, Yota Kawasaki, Ken Sasaki, Michiyo Higashi, Hiroaki Kobayashi, Takaaki Arigami, Takao Ohtsuka

**Affiliations:** 1 Department of Digestive Surgery, Graduate School of Medical and Dental Sciences, Kagoshima University, Kagoshima, Kagoshima, Japan; 2 Department of Pathology, Graduate School of Medical and Dental Sciences, Kagoshima University, Kagoshima, Kagoshima, Japan; 3 Department of Obstetrics and Gynecology, Graduate School of Medical and Dental Sciences, Kagoshima University, Kagoshima, Kagoshima, Japan

**Keywords:** perineal tear, perineal laceration, robot-assisted surgery, rectal neuroendocrine tumor, ultra-low anterior resection

## Abstract

**INTRODUCTION:**

Surgical repair of severe perineal tears is required immediately postpartum. Owing to their low prevalence, the post-treatment course of severe tears is not well known. Herein, we report a rare case of a young woman who underwent robot-assisted curative resection with anal preservation for a rectal neuroendocrine tumor (NET) incidentally discovered following a severe perineal tear.

**CASE PRESENTATION:**

A 29-year-old woman experienced a severe perineal tear during the first vaginal delivery, which led to the incidental discovery of a 20-mm rectal NET. Four months after the perineal tear, the gynecology and digestive surgery teams ensured that the tear wound had completely healed and anal function was preserved. The patient underwent robot-assisted ultra-low anterior resection with lymph node dissection. The procedure was successfully completed, preserving anal function, and histopathology confirmed an NET (G2, pT2N2aM0, pStage IIIB). The patient recovered smoothly and was discharged on the seventh postoperative day.

**CONCLUSIONS:**

Rectal surgery after severe perineal tears may be associated with scarring and fibrosis around the rectum, and precautions should be taken at the time of rectal dissection. Depending on the tumor condition, it may be advisable to perform rectal surgery several months after the tear rather than immediately after treatment for the tear.

## Abbreviations

NET
: neuroendocrine tumor
UICC
: The Union for International Cancer Control

## INTRODUCTION

Perineal tear is a complication that occurs when the baby’s head passes through the vagina.^1,2)^ It is classified as a minimal first-degree to severe fourth-degree injury, depending on the extent to which the damage has developed. Fourth-degree perineal tears, in which the vaginal wall is damaged from the external anal sphincter to the rectal mucosa, are rare. Although surgical repair is required immediately postpartum in severe perineal tears, the impact of rectal surgery after a severe perineal tear is not clear.

Herein, we present a rare case of rectal neuroendocrine tumor (NET) incidentally discovered following a fourth-degree perineal tear, which was subsequently treated with robot-assisted ultra-low anterior resection.

## CASE PRESENTATION

**Fig. 1:**
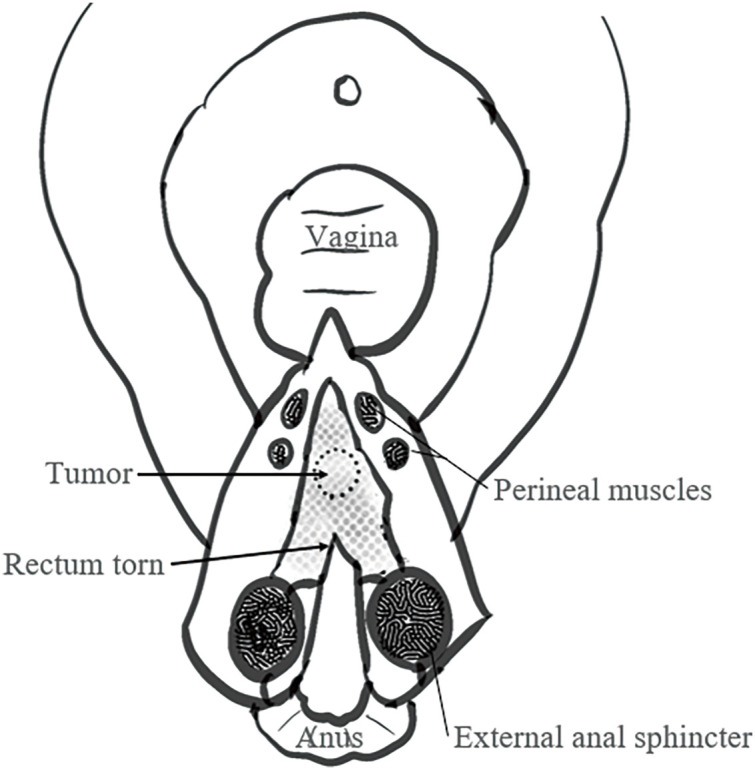
Illustration of the relationship between a fourth-degree perineal tear and tumor position.

**Fig. 2:**
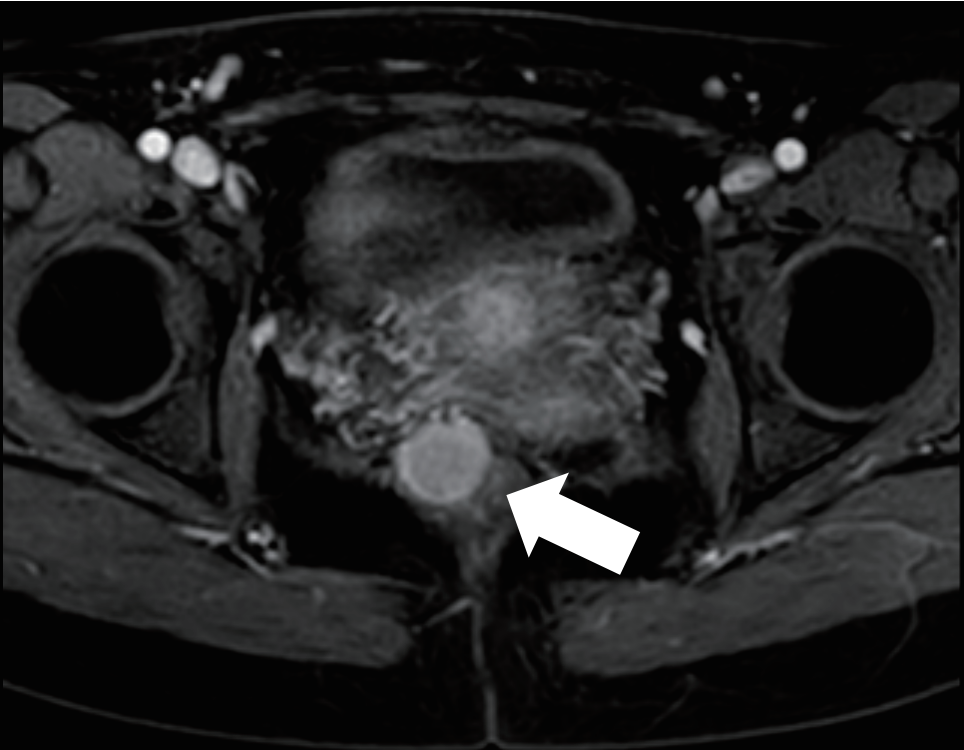
Pelvic MRI findings. The rectal tumor (white arrow) is in the form of a 20-mm submucosal tumor in the lower rectum. MRI, magnetic resonance imaging

**Fig. 3:**
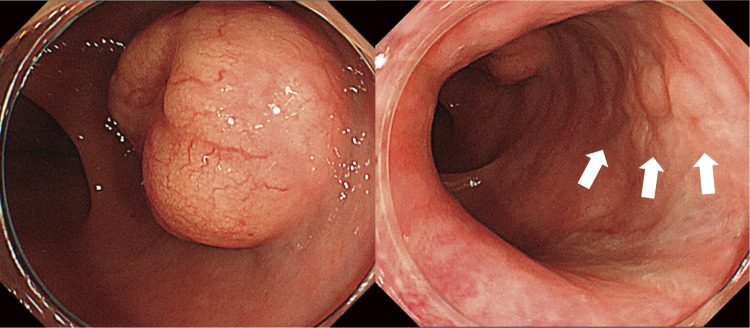
Colonoscopy image. A submucosal tumor with a central depression is identified on the anterior wall of the lower rectum, and scarred rectal mucosa (white arrows) due to a previous fourth-degree perineal laceration is observed distal to the tumor.

**Fig. 4:**
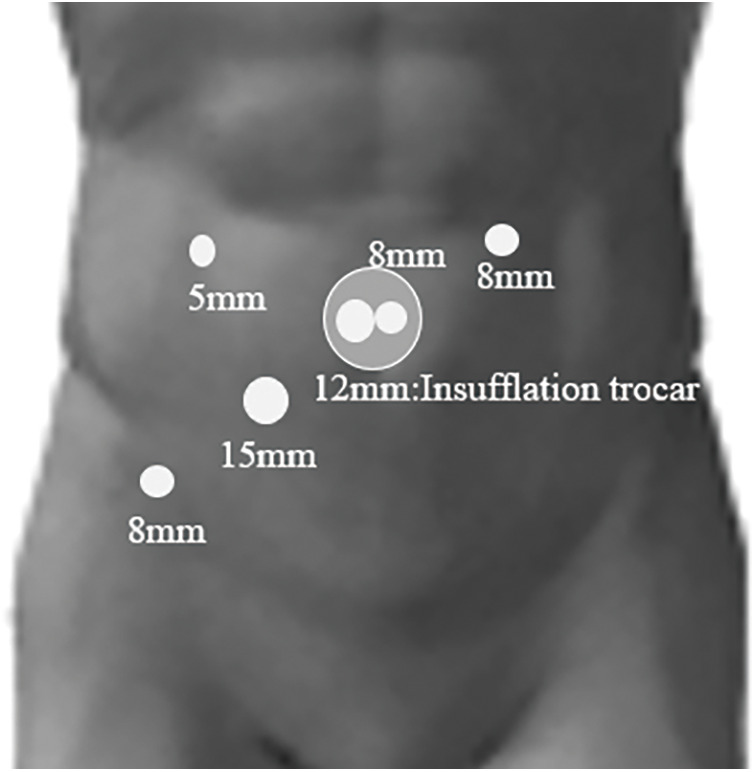
Robotic trocar placement.

**Fig. 5:**
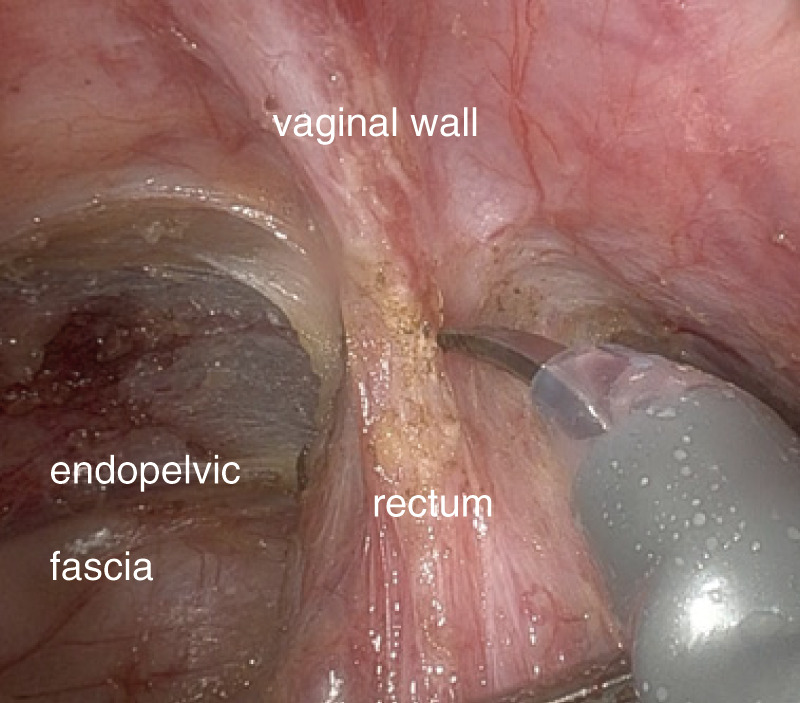
Representative intraoperative image.

**Fig. 6:**
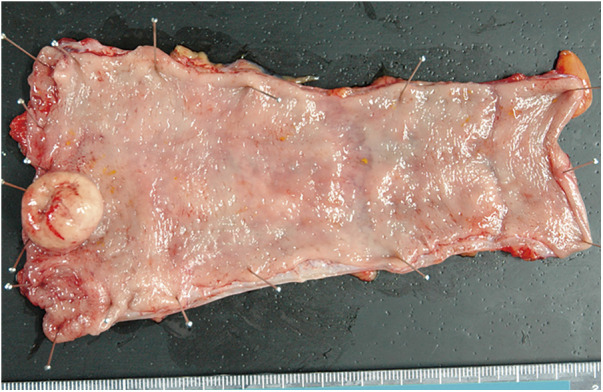
Macroscopic findings of the resected specimen.

A 29-year-old woman experienced a fourth-degree perineal tear during the first vaginal delivery. The patient had no relevant medical or family history. Although the patient had no physical symptoms, gynecological examination revealed a rectal mass during treatment (**Fig. 1**). The perineal tear was repaired transperineally, with an uneventful postoperative course. Laboratory examinations upon admission did not reveal any specific findings. One month after diagnosing the perineal tear, the rectal tumor appeared as a 20-mm submucosal tumor in the lower rectum on pelvic magnetic resonance imaging (**Fig. 2**). No obvious lymphadenopathy was observed. Colonoscopy revealed a 20-mm submucosal tumor with a central depression located on the anterior wall of the lower rectum, 4 cm from the anal verge, and scarred rectal mucosa on the anal side of the tumor, indicative of a fourth-degree perineal tear. The scar was observed 3 cm above the anal verge and 1 cm on the anal side of the tumor. (**Fig. 3**). A rectal biopsy of the tumor confirmed the diagnosis of NET G1, and the patient was referred to the Department of Digestive Surgery for surgery. Contrast-enhanced computed tomography of the chest and abdomen revealed no lymph node enlargement or distant metastases. Due to the patient’s lactation status, positron emission tomography-computed tomography and somatostatin receptor scintigraphy were not performed. The gynecology and digestive surgery teams ensured that the tear wound had completely healed and anal function was preserved, as evaluated by clinical symptoms, such as the absence of fecal incontinence and maintenance of an anal tone. Consequently, we determined that proceeding with rectal surgery 4 months after the injury was appropriate. The preoperative diagnosis was rectal NET G1, 20 mm, cT2N0M0, Stage IIA according to the 8th edition of the Union for International Cancer Control (UICC) TNM classification. Preoperative preparation included of mechanical bowel preparation with magnesium citrate and sodium picosulfate, and oral antibiotic bowel preparation with kanamycin and metronidazole. The patient underwent robot-assisted ultra-low anterior resection 4 months after being diagnosed with a perineal tear (**Fig. 4**). The inferior mesenteric artery lymph nodes (station 253) were dissected while preserving the left colic artery. The superior rectal artery was clipped at its origin and D3 lymph node dissection was performed. Total mesorectal excision was performed for rectal mobilization. During anterior rectal wall dissection, fibrous adhesions were observed between the vagina and anterior rectal wall beyond the tumor site. These adhesions were thought to be associated with rectal injury sustained during perineal tears. Therefore, we decided to leave this area for the final dissection, and the surrounding tissues were carefully separated. The fibrous adhesions were carefully dissected using monopolar cautery, successfully separating the rectal wall from the vaginal wall without injury (**Fig. 5**). The rectum was mobilized to the surgical anal canal level, confirming the absence of residual tumor or rectal injury on digital examination. The rectum was transected in a single firing by the assistant surgeon using a 60-mm laparoscopic powered linear stapler through the third robotic trocar. Indocyanine green fluorescence imaging was used for specimen extraction (**Fig. 6**), after which anastomosis was performed using a 25-mm circular stapler. Furthermore, to reinforce the anastomotic sites, two staple intersection points were sutured in Z-sutures using absorbable materials with robotic assistance, and a leak test confirmed the absence of air leakage. In addition, diverting stomas were not created, and a trans-anal drain was used postoperatively to monitor for any possible leakage or complications from the anastomosis. The patient started water intake on the postoperative Day 1 and began dietary intake on the postoperative Day 3. The trans-anal drain was removed on postoperative Day 5 when no signs of leakage were observed. The postoperative course was uneventful, and the patient was discharged on the seventh postoperative day. Histopathological examination revealed NET G2 (Ki67 index = 5%), pT2 (MP)N2aM0, pStage IIIB (UICC TNM classification, 8th edition). At 6 months postoperatively, the patient exhibits no issues with anal function, no signs of recurrence, and is progressing well.

## DISCUSSION

Perineal tears occur in up to 90% of nulliparous women, with the majority being first- or second-degree tears.^3)^ The incidence of fourth-degree perineal tears is rare, ranging from 1.1% to 5.4%; therefore, the post-treatment course of severe tears is not well known. We searched the PubMed database for studies published in English using the terms “perineal tear” or “perineal laceration” and “rectal surgery,” but we could not find reports on rectal surgery after severe perineal tears. Therefore, focusing on the present case, we discuss the wound healing process after the treatment of severe perineal tears and the optimal timing and surgical approach for rectal resection after perianal injuries.

Third-degree perineal tears involve partial damage to the external anal sphincter, and fourth-degree perineal lacerations extend from the vaginal wall to the external anal sphincter and then to the rectal mucosa, which require immediate suturing after delivery.^1)^ If not properly treated, perineal tears can lead to anal sphincter dysfunction and fecal incontinence. If suturing fails, fistula formation can occur. In this patient, the postoperative course of the perineal tear was favorable; fortunately, the external anal sphincter was not completely torn, preserving anal function. In addition, during the 4-month wait after the injury, the rectum and vagina had healed, and ultra-low anterior resection could be performed. Before rectal surgery, the extent and location of the injury, tumor location, and anorectal function should be assessed to determine the surgical strategy.

The optimal time for rectal surgery following a perineal injury should be after complete healing of the injury. However, the time required for a wound to heal varies, and many factors influence the healing process. Simple wounds often heal within 30 days, while complex or contaminated wounds may become chronic.^4,5)^ In this patient, the tumor was diagnosed as an NET on biopsy; therefore, we waited for the rectal injury to heal perfectly. Rectal NETs are rare tumors that arise from neuroendocrine cells in the rectum and typically exhibit slow growth.^6)^ The prognosis of rectal NETs is generally better than that of other gastrointestinal NETs, with a 5-year survival rate of 87%–94% in non-metastatic cases. However, patients with lymph node metastasis have a 5-year survival rate of 54%–73%, and those with distant metastasis have a poor prognosis, with a 5-year survival rate of 15%–30%.^6–8)^ The incidence of rectal NETs has been increasing, with a reported incidence rate of 0.32 per 100000 population.^6,9,10)^ Treatment guidelines recommend local excision of tumors <1 cm, while tumors >1 cm require curative resection with lymphadenectomy.^11)^ In the present case, the tumor was 2 cm in diameter and warranted rectal resection with lymph node dissection.

Robot-assisted surgery for rectal cancer is safe and now an important surgical technique for rectal procedures.^12,13)^ Saito *et al*. reviewed 27 cases of robot-assisted surgery for rectal NETs performed at a single institution between 2011 and 2017 and reported favorable outcomes with anal preservation and no severe postoperative complications for low-lying tumors.^14)^ Robotic surgery offers advantages such as precise movements, tremor reduction, and high-resolution three-dimensional imaging, allowing for more meticulous surgery in the confined space of the pelvic cavity than traditional laparoscopic surgery.^14)^ In our patient, robotic surgery facilitated safe dissection and resection of the rectal NET, even in the presence of adhesions due to previous rectal injury.

## CONCLUSION

Rectal surgery after a severe perineal tear may be associated with scarring and fibrosis around the rectum, and precautions should be taken during rectal dissection. We could safely perform rectal surgery after 4 months; however further research is required to determine the optimal timing for surgery, considering factors like the tumor condition.

## ACKNOWLEDGMENTS

We thank Editage (http://www.editage.com) for editing and linguistic proofreading of this manuscript.

## DECLARATIONS

### Funding

This study was supported by Grant-in-Aid for Scientific Research (KAKEN) 23K19835.

### Authors' contributions

KB drafted the manuscript.

KB, MW, NK, YH, DK, MS, YK, KS, and TA managed the perioperative course and collected the data.

MH contributed to the pathological study.

HK contributed to the gynecological study.

TO supervised the writing of the manuscript.

All authors discussed the content of the manuscript.

All authors read and approved the final manuscript.

### Availability of data and materials

Data availability does not applicable to this article as the datasets have not been generated or analyzed.

### Ethics approval and consent to participate

Not applicable.

### Consent for publication

Written informed consent was obtained from the patient for the publication of this article and any accompanying images.

### Competing interests

The authors declare that they have no competing interests.
